# Care for Vulnerable Populations with Chronic Liver Disease: A Safety-Net Perspective

**DOI:** 10.3390/healthcare11202725

**Published:** 2023-10-13

**Authors:** Mark C. Wang, Saroja Bangaru, Kali Zhou

**Affiliations:** 1 Department of Medicine, Keck School of Medicine, University of Southern California, Los Angeles, CA 90033, USA; markwang@usc.edu (M.C.W.); bangaru@usc.edu (S.B.); 2 Los Angeles General Medical Center, Los Angeles, CA 90033, USA; 3 Research Center for Liver Diseases, Keck School of Medicine, University of Southern California, Los Angeles, CA 90033, USA

**Keywords:** end-stage liver disease, health disparities, safety-net health system, health services research, socioeconomic disparities

## Abstract

Safety-net hospitals (SNHs) and facilities are the cornerstone of healthcare services for the medically underserved. The burden of chronic liver disease—including end-stage manifestations of cirrhosis and liver cancer—is high and rising among populations living in poverty who primarily seek and receive care in safety-net settings. For many reasons related to social determinants of health, these individuals often present with delayed diagnoses and disease presentations, resulting in higher liver-related mortality. With recent state-based policy changes such as Medicaid expansion that impact access to insurance and critical health services, an overview of the body of literature on SNH care for chronic liver disease is timely and informative for the liver disease community. In this narrative review, we discuss controversies in the definition of a SNH and summarize the known disparities in the cascade of the care and management of common liver-related conditions: (1) steatotic liver disease, (2) liver cancer, (3) chronic viral hepatitis, and (4) cirrhosis and liver transplantation. In addition, we review the specific impact of Medicaid expansion on safety-net systems and liver disease outcomes and highlight effective provider- and system-level interventions. Lastly, we address remaining gaps and challenges to optimizing care for vulnerable populations with chronic liver disease in safety-net settings.

## 1. Introduction

The mission of safety-net hospitals (SNH) and facilities in the United States (US) is to provide comprehensive and critical healthcare services to individuals regardless of their ability to pay. For those with chronic liver disease, there is a greater reliance than ever on these institutions, as the burden of cirrhosis and its complications has increased over the past two decades, particularly among populations living in poverty who are under- or un-insured. This was the case when hepatitis C (HCV) was the dominant liver disease etiology in the US and remains the case as alcohol-associated liver disease (ALD) and metabolic dysfunction-associated steatotic liver disease (MASLD) come to the forefront. These safety-net populations with liver disease—comprising low-income racial/ethnic minorities and immigrants in both urban and rural regions—face substantial obstacles in accessing high-quality healthcare and in turn experience many disparities across disease conditions, which is increasingly recognized to be the consequences of negative structural and social determinants of health.

In this narrative review, we describe the increasing burden of chronic liver disease and cirrhosis-related complications in the low-income populations served by SNHs, highlight barriers to care and disparities in liver-related outcomes, and provide an overview of patient-, provider-, and system-level interventions in the delivery of SNH liver disease care. A focused perspective on the impact of Medicaid expansion and policies on both liver disease outcomes and safety-net systems is also presented. We conclude with an overview of knowledge gaps and future directions for research and policy.

## 2. Overview of Safety-Net Care in the United States

**Figure 1:**
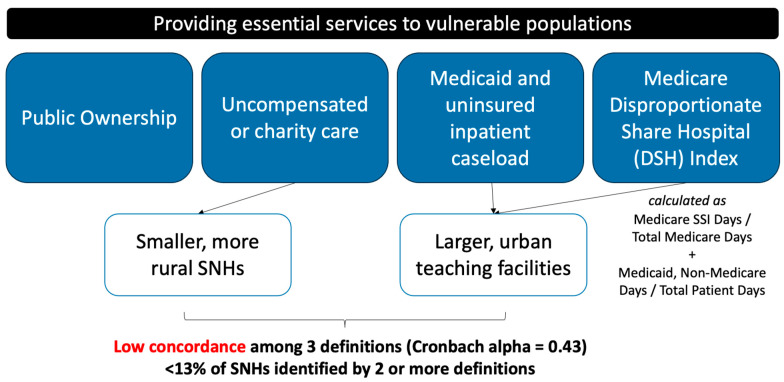
Controversy in definition of a safety-net health system. SSI = social security income.

The Institute of Medicine defines the safety net in healthcare as “those providers that organize and deliver a significant level of health care and other needed services to uninsured, Medicaid and other vulnerable patients” [1]. This definition casts a wide net, and operationally, the type of hospitals that are considered safety-net systems are heterogeneous in nature. The spectrum spans from public hospitals that provide essential services and academic teaching centers with a mission to serve the underserved to private hospitals that fill the role when public hospitals are not proximately available. At present, there is no single consensus definition that is used in policymaking or research and each definition has inherent strengths and shortcomings [2]. Some possible definitions include facilities that are publicly owned, those that provide uncompensated or charity care, or those with a high caseload of Medicaid and uninsured; few incorporate external social risk scores [3,4,5] (see Figure 1). One commonly used definition in research is the Medicare Disproportionate Share Hospital (DSH) index, calculated with data obtained from the Centers for Medicare & Medicaid Services cost reports. A study comparing several definitions found low concordance across them (13% identified by two of three definitions), with uncompensated care accounting for a larger proportion of smaller rural hospitals while DSH and a caseload mix indicated larger teaching facilities [6]. Further complicating cross-study comparisons are the multitude of different thresholds or binary categories used to designate safety-net status. When defined as top quartile of Medicaid and uninsured discharges per state, SNHs were more likely than non-SNHs to be teaching hospitals with a large number of inpatient beds and located in metropolitan centers [7].

Regardless of the definition used, there are general characteristics of the population served—and barriers to care—that are consistent across safety-net systems. First, a higher proportion of safety-net populations are racial and ethnic minorities, more so in urban areas (60% non-White in safety-net hospitals vs. 40% in non-SNHs) [8]. These individuals are often less educated with lower household incomes, living at or below the federal poverty level [8]. Immigrants are also more likely to access care at safety-net facilities, which specifically serve as a critical touchpoint for medical services to undocumented persons [9]. Per its definition, safety-net systems provide services to the Medicaid-insured—a substantial subsection of the nation as over 56 million individuals were beneficiaries of the program in 2019 [10]—and the 26 million that remain uninsured [11]. Related to these social determinants of health, concerns about finances, lack of transportation or stable housing, and challenges in navigating a complex healthcare system are all structural barriers to seeking care for safety-net populations [12]. Compounding the disadvantage is the fact that SNHs typically operate with smaller budgets and more limited resources, which serve as unique constraints to implementing measures to improve quality and equity [13,14], and have led to smaller incremental gains in performance measures [15].

## 3. Epidemiology of Chronic Liver Disease in Safety-Net Populations

**Figure 2:**
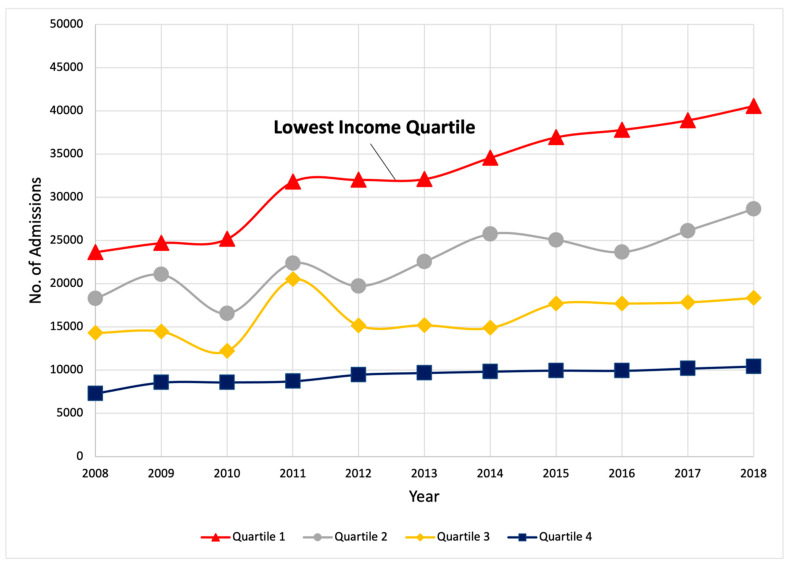
Trends in inpatient hospitalizations for cirrhosis among government-owned public hospitals by income quartile. Data from the National Inpatient Sample (NIS) dataset, which is part of the Healthcare Cost and Utilization Project (HCUP) and sponsored by the Agency for Healthcare Research and Quality. Cirrhosis was defined using the International Classification of Diseases, Ninth Revision (ICD-9) (1 January 2005 through 30 September 2015) diagnosis codes 571.2, 571.5, and 571.6 or International Classification of Diseases, Tenth Revision (ICD-10) (1 October 2015 through 31 December 2018) diagnosis codes K70.3, K70.30, K70.31, K74.0, K74.00, K74.01, K74.02, K74.1, K74.2, K74.3, K74.4, K74.5, K74.6, K74.60, and K74.69. Income quartiles were established by the NIS as the median household income of the patient’s zip code sorted into quartiles (Q) defined by Claritas annually (www.hcup-us.ahrq.gov, accessed on 30 August 2023). Yearly estimates of hospitalizations were generated using HCUP discharge trend weights.

Chronic liver disease and cirrhosis are common in the US, accounting for 400,567 emergency department visits and 280,645 hospital admissions in 2018, according to the Healthcare Cost and Utilization Project (HCUP) [16]. The burden is estimated to be highest and rising among low-income populations and areas. According to National Health and Nutrition Examination Survey (NHANES) data, cirrhosis patients are disproportionately impoverished compared to those without cirrhosis, as measured via annual incomes of ≤USD 20,000 (50.5% vs. 32.5%, *p* = 0.0005) [17]. Inpatient admissions for cirrhosis among US public hospitals have increased year-after-year, with the highest frequency and greatest gains in the lowest-income quartile (Figure 2). The low-income group is one of few groups remaining with rising rather than plateauing liver cancer incidences [18]. Age-adjusted mortality from cirrhosis has also increased in all geographic regions since 2008, being highest in nonmetropolitan areas and counties with increased poverty and a lack of insurance [19]. Indeed, counties with the smallest improvement in economic prosperity over time have been linked to the largest increases in liver-related mortality [20], highlighting the important relationship between population-level persistent poverty and the chronic liver disease burden.

The liver disease etiologic profile of safety-net patient populations may depend on the clinical context, though ALD and HCV are leading conditions. Among the hospitalizations in five different safety-net hospitals across the country, ALD alone was most common at 28–49% [21,22]. This was followed by viral hepatitis alone at 15–43%, combined viral hepatitis and ALD at 9–43%, MASH/MASLD at 2–8%, and other/cryptogenic etiologies at 2–13% [21,22]. However, because these statistics are based on inpatient data, they may overrepresent etiologies associated with rapid disease progression and acute decompensation, while underrepresenting etiologies with an indolent course. ALD was also the dominant etiology (53.7%), followed by HCV (27.1%), in a multicenter SNH cohort of outpatient referrals for liver transplantation [23].

## 4. Chronic Liver Disease Disparities in Safety-Net Settings

**Figure 3:**
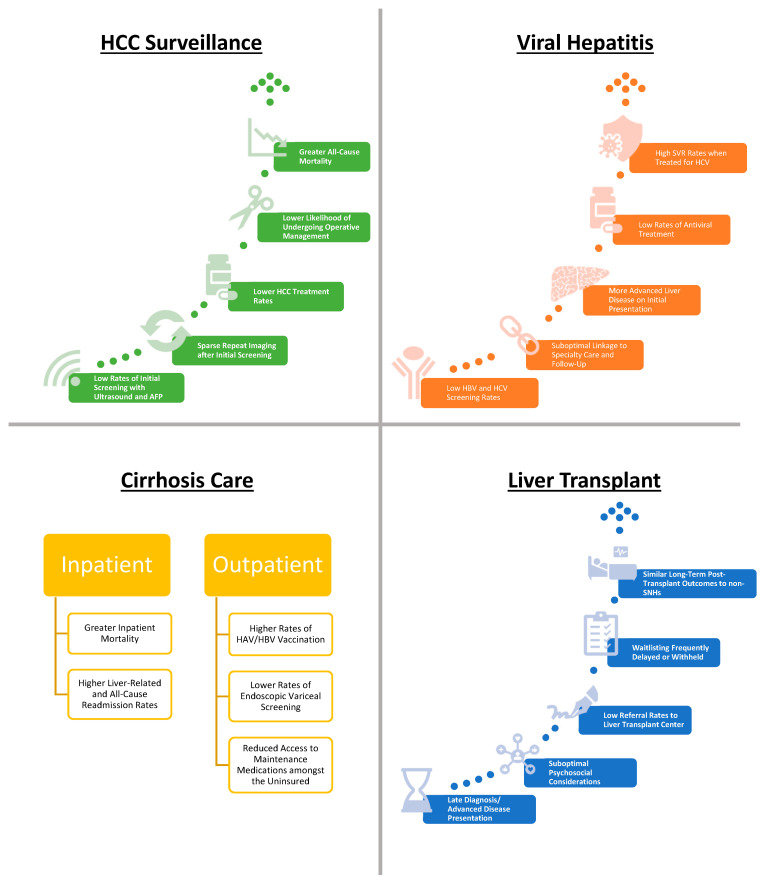
Disparities in care and outcomes among safety-net patients with liver disease conditions.

The following sections highlight presentation and outcome disparities experienced within safety-net systems and among safety-net populations for common liver disease conditions (see Figure 3 for a graphical summary). A comprehensive literature search was performed, and pertinent studies are highlighted with the prioritization of more recent publications.

### 4.1. Metabolic- and Alcohol-Associated Steatotic Liver Diseases

Both MASLD and ALD are highly prevalent and increasing in safety-net populations. Similar to the general US population, over a third of a cohort of >45,000 safety-net patients were obese, with a higher body mass index (BMI) associated with elevated transaminases. Interestingly, the correlation between BMI and transaminase elevation was stronger in safety-net patients without diabetes compared to those with diabetes, supporting the need to expand MASLD screening beyond traditional high-risk groups [24]. A provider survey found that only 31% of providers in managed care, academic, and safety-net settings found MASLD to be an important clinical diagnosis; compared to those in other settings, safety-net providers were the least likely to refer MASLD patients to a dietician (55% vs. 80% and 92%, respectively) and also less likely to refer them to specialists [25].

From 2011 to 2017, the percentage of alcohol-induced hepatitis hospitalizations with Medicaid as the primary payer increased from 21.5% to 32.5% [26], with inpatient diagnoses of ALD and alcohol-induced hepatitis being more common among those with Medicaid than those with Medicare [27]. According to an analysis of the National Inpatient Sample, patients treated at high-safety-net-burden hospitals were at a significantly greater risk of inpatient mortality due to alcohol-associated cirrhosis compared to those treated at low-safety-net-burden hospitals. The mortality trend was similar for alcohol-induced hepatitis hospitalizations, but the difference was not statistically significant [28]. Finally, as with other diseases, care for steatotic liver diseases was disrupted by the recent COVID-19 pandemic. In a survey of safety-net hepatology clinic patients with MASLD or ALD, 51% reported decreased physical activity, and 34% showed weight gain, mostly as a result of low physical activity and heavy alcohol use during the pandemic. Participants were also queried about telemedicine visits. Only 62% of patients were very satisfied with their visit, with Hispanic patients reporting lower satisfaction—highlighting an area for improvement as the utilization of telemedicine continues to expand [29].

### 4.2. Surveillance and Outcomes for Hepatocellular Carcinoma

There are suboptimal rates of guideline-concordant screening and surveillance for HCC (ultrasound and alpha-fetoprotein every 6 months) in the US for all cirrhotics, but they are lowest in those of a lower socioeconomic status [30]. Only one in four patients underwent HCC surveillance via any imaging type in the year leading up to diagnosis in one SNH [31]. Low repeat surveillance imaging may be a bigger problem as opposed to a lack of initial imaging. In a Northern California safety-net population, the majority (74.8%) of cirrhotic patients underwent initial screening within one year of diagnosis, but only 47.6% (95% CI 41.4–51.2%) had follow-up surveillance imaging within the next year [32]. Both safety-net and rural settings have been negatively associated with surveillance, especially compared to academic settings, suggesting that system-level factors likely play a role [33]. Provider-level barriers including lack of resources, decreased access to specialty referrals, time constraints, scheduling issues, and difficult communication with patients, as well as patient-level barriers such as financial barriers, transportation barriers, insurance concerns, and limited adherence are all more common among safety-net populations [33,34].

Among safety-net patients, a lack of surveillance has been associated with more advanced HCC at diagnosis and worse overall survival, particularly among the uninsured [35,36,37]. However, there may not be a difference in tumor stages between SNHs and other hospital types [35,36]. Disparities in the outcomes for safety-net populations extend across the HCC continuum of care. In a study of 267 treatment-eligible safety-net patients, 38% did not receive HCC treatment, and 30% of those treated experienced therapeutic delays lasting at least 3 months [38]. Similarly, in the Texas Cancer Registry, HCC patients diagnosed at SNHs, in accordance with the DSH index, compared to those at non-SNHs, underwent lower treatment utilization (OR, 0.64; 95% CI, 0.57–0.73) and were less likely to receive curative therapy (OR, 0.51; 95% CI, 0.40–0.66), resulting in worse overall survival [39].

### 4.3. Screening and Follow-Up of Chronic Viral Hepatitis

Safety-net patients also experience disadvantages at multiple stages of the viral hepatitis care continuum, starting with screening. At one large SNH in California, the risk-based screening rates for hepatitis B (HBV) and HCV were only 25.1% and 30.9%, respectively [40], with lower rates among Asians, Hispanics, and foreign-born patients [40,41]. Safety-net patients undergoing rituximab therapy also had low rates of pre-initiation HBV screening at 14.7% in an earlier time period (2006–2009), though significant improvement was seen by the period 2013–2015 at a rate of 85.9% with increased awareness and quality improvement initiatives [42]. Universal screening for both HBV and HCV is now endorsed by the Centers for Disease Control [43,44]; the impact of these guideline changes needs further study.

Downstream from screening, follow-up testing and linkage to care for viral hepatitis has been relatively poor amongst safety-net populations. In a retrospective analysis of 600 safety-net patients with chronic HCV between 2002 and 2018, only 57.7% were seen by HCV specialists within a year of diagnosis, though the vast majority (91.6%) were followed-up after the initial visit [45]. In terms of follow-up laboratory testing, data from a San Francisco safety-net system found that 22.9% of HCV antibody-positive patients did not receive further testing for HCV RNA [46]. For chronic HBV, Hispanic safety-net patients, in particular, have been associated with lower rates of follow-up testing and linkage to care compared to those of Asian patients [47]. Together, lower rates of screening and linkage to care may lead to higher frequency of cirrhosis at presentation [48].

Fortunately, treatment for viral hepatitis is highly effective. Across multiple US SNHs, real-world studies report high sustained virologic response (SVR) rates for direct-acting anti-HCV (DAA) treatment in underserved populations with significant barriers to healthcare [45,46,49,50,51,52]. Antiviral treatment for chronic HBV in safety-net settings has also been associated with a reduced risk of cirrhosis as well as morbidity and mortality [53]. However, access to these treatments can be a barrier and follow-up among treatment-initiated remains a concern. According to a multicenter study of SNH patients with HBV, less than 50% of treatment-eligible patients received therapy [54]. Reported treatment rates for HCV are estimated to be at 70% at best, though a significant proportion may defer treatment due to comorbidities [49,51]. After starting HCV treatment, loss-to-follow-up and non-completion rate range from 22 to 25% [55], and the completion of post-SVR HCC surveillance remains inadequate [56]. Some safety-net demographics found to have lower rates or delays in HCV treatment include those of a female sex, black people, Hispanics, and those with governmental or no insurance [45,57,58].

### 4.4. Cirrhosis Care and Liver Transplantation

The safety-net setting presents unique challenges for patients with cirrhosis. According to a study of cirrhosis patients admitted for liver-related decompensations in four SNHs across the country, inpatient mortality ranged from 4 to 13%, and up to a quarter were readmitted within 30 days [21]. Comparing hospitals with different levels of safety-net burdens (based on the proportion of Medicaid and uninsured patients), high- and medium-burden hospitals had 5% greater in-hospital mortality compared to that in low-burden hospitals for cirrhosis-related hospitalizations [59]. Data from the North American Consortium for the Study of End-Stage Liver Disease (NACSELD) cohort of hospitalized patients with cirrhosis suggested that uninsured patients were less likely to utilize cirrhosis-related medications, and insurance status but not race and ethnicity was independently associated with length of stay, acute-on-chronic liver failure, and death [60]. In the outpatient setting, SNHs had higher rates of HCC surveillance and hepatitis vaccination compared to those in an academic and Veterans Affairs practice in one study, but also had the lowest rates of endoscopic variceal screening (43% for the SNH vs. 58% for the VA practice vs. 75% for the faculty clinic) and liver transplant discussions [61].

Liver transplantation (LT) is lifesaving for patients with complications of cirrhosis but is a difficult and complex process for safety-net populations to navigate. Sociodemographic disparities in access to LT are frequently reported, including lower referral and higher MELD upon listing among racial/ethnic minorities and the underinsured [62,63]. In a recent multicenter study of 521 patients at three SNHs in the Western US, fewer than one in three patients with MELD ≥ 15 were referred to a local transplant center over a median of 2.5 years of follow-up. Predictors of non-referral included male sex, black race/ethnicity (compared to Hispanic/Latinx ethnicity), unstable housing, and lack of insurance. Referral was most commonly withheld due to psychosocial considerations such as recent alcohol use, insurance, or limited social support [23]. Among the one-third referred for transplant in the multicenter cohort above, 73.1% were evaluated, 35.2% were listed, and 19.3% were transplanted [23]. Drop-offs were also seen among patients with HCC in SNHs, with a mere 5% being wait-listed and 1.3% being transplanted [64]. Post-transplant, patients had higher perioperative mortality at medium- and high- safety-net-burden hospitals (vs. low-safety-net-burden-hospitals) (4.5% and 5.2%, respectively, vs. 2.9%) but showed no differences in readmission rates and 3-year overall and graft survival [65]. For undocumented immigrants, a special population within the safety-net context, access to LT varies widely by geography; a larger proportion are accounted for in California, New York, Illinois, and Minnesota relative to each of their estimated undocumented populations [66]. There has been no difference observed in post-transplant graft survival and mortality after transplantation [66].

## 5. Interventions to Improve Care Delivery in Safety-Net Settings

To address some of the disparities highlighted above, many studies have focused on interventions to improve care delivery to vulnerable populations with liver disease within safety-net systems (see Table 1). The strategies commonly studied include (1) educational programs, (2) patient navigation, and (3) mailed outreach. Targeted patient or provider education has shown success. A retrospective study evaluated the effectiveness of a formal HCV education class given by liver specialists to primary care physicians (PCPs) and demonstrated a reduction in time to the initiation of HCV treatment (median 136 vs. 284 days, *p* < 0.0001) and an independent association between HCV education and SVR (OR, 3.0; 95% CI, 1.1–7.9; *p* = 0.03) [67]. When combined with an electronic medical record (EMR)-enabled best practice alert and expanded HCV treatment capacity, patient and provider education dramatically increased HCV screening among eligible baby boomers, from 10.1% to 34.6% (*p* < 0.0001) [68]. Similarly, for chronic HBV care, an in-person, language-concordant patient education session improved rates of appropriate HBV follow-up, monitoring, and treatment uptake [69].

Among safety-net populations, patient navigators have the potential to improve the completion of screening, linkage to care, and retention within healthcare systems [80]. For example, adherence to HCC screening within an established HCV treatment clinic was much higher than previously reported at 94%, 75%, and 74% for first, second, and third imaging tests, respectively, when navigators were used for scheduling imaging appointments and tracking compliance [77]. More recently, a comparative study of patient navigation on the linkage of HCV-infected baby boomers to subspecialty care found that patient navigation increased the odds of linkage to care by four-fold and treatment initiation by three-fold within 6 months [79].

Mailed outreach programs within safety-net healthcare systems have increased success in recommended screening uptake. For HCC surveillance, Singal et al. demonstrated that mailed invitations for HCC screening US plus patient navigation (47.2%) or mailed invitations alone (44.5%) yielded higher screening rates compared with the rates for those who received usual care (24.3%) (*p* < 0.001 for both comparisons) [72]. An extension of this study evaluating continued HCC surveillance over an 18-month period found the highest rates of ongoing surveillance in the outreach plus navigation group [75]. Notably, these gains were observed at the safety-net hospital but not at a tertiary care center [78], suggesting the potential for a higher impact of patient navigation and mailed outreach in resource-limited settings. Mailed outreach has also effectively increased HCV screening rates within safety net systems (17.4% vs. 9.8%, *p* < 0.001) at 6 months post-randomization [76].

Additional innovations within safety-net systems are worth mentioning. In a SNH in Atlanta, establishing a HCC multi-disciplinary management team increased HCC treatment rates (59% vs. 41%; *p*  =  0.03) and median overall survival (30.7 vs. 4.9 months; *p*  < 0.001) [31]. The use of a voice messaging system to notify ordering and downstream treating physicians of a new HCC diagnosis significantly reduced the time from diagnosis to clinic contact and treatment (2.2 vs. 5.5 months; *p* = 0.005) [70]. Lastly, a prospective pilot study on co-locating HCV screening services at the time of endoscopy demonstrated the feasibility and productivity of utilizing existing patient encounters in linking additional HCV-infected patients to treatment [73].

## 6. Impact of Medicaid Policies on Safety-Net Institutions

On 1 January 2014, a Medicaid expansion (ME) policy was enacted under the Affordable Care Act (ACA) in which states had the option to extend Medicaid coverage to most non-elderly adults with an income of up to 133% of the federal poverty line. Under this policy, 30 states expanded Medicaid in 2014. In fact, ME led to one of the largest gains in in health insurance for non-elderly adults in the United States and extended health insurance to >20 million previously uninsured patients [81]. This policy change had many favorable impacts on health measures and outcomes, including increased PCP and outpatient appointments, a reduction in annual out-of-pocket medical spending, reductions in skipped medications due to costs, and decreased emergency room (ER) utilization [81]. Importantly, several studies have shown reductions in mortality in the post-ME period [82,83,84], although these gains vary greatly by state [84], with greater state-level reductions among women and non-Hispanic black residents in expansion states [84].

Various aspects of liver-related care have improved since ME. With regards to liver disease-related mortality, 8.3 (95% CI, 1.6–15.1) fewer liver-related deaths per 1,000,000 adult residents per year occurred in ME states compared with what would have been expected to occur if those states followed the same trajectory as non-ME states [85]. Cirrhosis-related hospitalization rates, complications of cirrhosis, readmissions, and costs were all lower in ME states when compared to those in non-ME states [86]. With respect to HCC care, patients in ME states were more likely to be diagnosed with early HCC, more likely to receive curative therapy, and less likely to die (HR = 0.68; 95% CI = 0.54–0.86) [87]. A 2017 UNOS study found that Medicaid enrollment increased by 4% among LT candidates in ME states, and one-quarter of transplant centers experienced a ≥10% increase in the proportion of LT candidates using Medicaid [88], though the absolute number and demographics of patients listed for LT did not change in participating states in the post-expansion period.

There is some data to suggest that minority groups have not benefitted equally from the ME policy with respect to LT, though this remains to be characterized further [89]. In addition, some studies have investigated heterogeneity in the effects of the ME policy in relation to the relative leniency or restrictiveness of state-specific Medicaid policies. For alcohol use disorder, there has been a differential uptake of early LT for ALD among restrictive vs. non-restrictive Medicaid policy states [90], suggesting that the standardization of state-based policies may increase LT equity. When examining the interaction between expansion status and state-specific leniency of Medicaid HCV coverage, improvements in end-stage liver disease (ESLD) mortality and waitlisting-to-death ratios were seen with Medicaid expansion but so was a leniency of HCV coverage [91]. In California, automatic eligibility for full-scope Medicaid coverage starting in May 2022 for all adults aged 50 or older regardless of immigration status [92] has simplified insurance access for undocumented individuals and may increase LT access for this important underserved group.

How has ME specifically impacted SNHs? One study examined the percentages of Medicaid inpatient days, percentage of Medicaid revenues, and uncompensated care costs as a percentage of total operating costs in the pre- and post-ME eras and found that ME had a significant favorable financial impact on SNH; ME states had larger Medicaid revenues and reduced uncompensated care costs, and these changes improved operating margins [93]. Another study comparing discharge data from SNHs in ME and non-ME states, pre- and post-expansion, found that expanded insurance coverage did not lead to systematic changes in the patient body receiving care at SNHs (i.e., a similar proportion of racial and ethnic minorities and patients residing in zip codes with higher poverty levels in all groups) and/or in the number of uninsured or Medicaid-insured hospitalizations [94]. Finally, investigations into the quality of SNH healthcare delivered as assessed via standard quality measures including patient-reported experiences, health care-associated infections, and 30-day readmission and mortality found no difference in these aspects, regardless of ME or non-ME status [95]. Based on these data, it appears that while ME increases insurance coverage for individuals, it has had a mixed impact on the delivery and quality of care in SNH systems.

## 7. Future Directions and Conclusions

One of the first steps to improving the delivery of care to SNH patients is to standardize the definition of a “safety-net” across research and policymaking in a manner that accurately captures the underlying population and resource constraints, such that funding can be appropriately allocated. Toward this end, there has been a call to action for a “sliding-scale” or continuous measure of disadvantage. Upstream policy changes that match SNH compensation to this sliding scale would be of great benefit. Next, addressing the well-documented disparities in SNH populations with liver disease requires sophisticated, comprehensive, and prospective assessments of social needs to facilitate a more targeted and effective implementation of social interventions. With respect to research, investigators should collect multilevel data (e.g., patients, SNH providers, SNH systems, communities, etc.) to investigate direct and indirect pathways and the interactions between such factors on liver disease care and outcomes. Lastly, we need to learn how best to implement strategies demonstrated to be effective in improving liver care delivery across different SNHs, including structured and integrated multi-disciplinary models, patient navigation services, and educational efforts. There is a gap in studies demonstrating the cost effectiveness and even potential cost savings incurred with these interventions; these studies would be helpful in promoting administrative buy-ins. Low-cost interventions that utilize existing healthcare visits or harness technology (e.g., electronic health records, text messaging, and telemedicine) may also be paths forward.

Challenges that remain to be addressed in SNHs include the following: (1) accessing the most vulnerable underserved populations (those who lack transportation means to hospitals, those who are homeless with no means of being contacted, etc.) who have multi-level barriers that impede healthcare delivery; (2) establishing programs (patient navigation services; multi-disciplinary teams) that are logistically and financially sustainable in the long term, to continue to address these care gaps; and (3) continually investing in and building the infrastructure needed to provide high-quality, cost-effective, and evidence-based care in safety-net systems. Achieving these goals will require a coordinated response from all safety-net stakeholders including but not limited to providers, hospital administrators, policymakers, researchers, and community advocates. Additionally, despite the many challenges ahead, the high prevalence of liver disease and its significant morbidity and mortality mean efforts to shore up the “safety-net” for our nation’s most vulnerable patients with chronic liver disease is a critical and worthwhile venture.

## Figures and Tables

**Table 1:** Summary of interventions on populations with chronic liver disease in safety-net health systems.

Study Author	Year	Intervention Type	Cohort Type	Study Design	Study Population	Main Findings
Lubega et al. [67]	2013	Formal Education	P	Pre- and post-intervention	Primary care providers	**Median # of days to initiating HCV treatment:** 136 (formal education group) vs. 284 (control), *p* < 0.0001 **Receipt of HCV education independently associated with SVR**: OR, 3.0; 95% CI, 1.1–7.9; *p* = 0.03
Mokdad et al. [70]	2016	Voice Messaging System	R	Pre- and post-intervention	Outpatients with HCC	**Shorter time from diagnosis to clinic contact:** 0.5 (post) vs. 2.9 (pre) months; *p* = 0.003 **Shorter time from detection to treatment:** 2.2 (post) vs. 5.5 (pre) months; *p* = 0.005 **Improved median survival:** 28.5 (post) vs. 15.7 (pre) months, *p* = 0.02
Konerman et al. [71]	2017	Electronic Health Record (EHR) Alert	R	Pre- and post-intervention	Baby boomers seen in PCP clinic	**Increased HCV screening rates:** 7.6% (6 months pre) to 72% (12 months post)
Singal et al. [72]	2017	Mailed Outreach +/− Patient Navigation	P	RCT	Patients with cirrhosis	**Increased HCC screening rates:** - Mailed outreach + navigation vs. usual care: 47.2% vs. 24.3%, *p* < 0.001 - Mailed outreach alone vs. usual care: 44.5% vs. 24.3%, *p* < 0.001 - Mailed outreach + navigation vs. mailed outreach alone: 47.2% vs. 44.5%, *p* = 0.25
Campbell et al. [73]	2018	Co-location of Visits	P	Descriptive and non-comparative	Baby boomers presenting for endoscopy	**High screening rates and linkage-to-care:** 84.2% of patients screened for HCV All those with confirmed infection linked to HCV clinic
Tran et al. [74]	2018	On-site Pharmacy (vs. Off-site)	R	Case control	Patients with HCV infection treated with DAA	**Increased likelihood of SVR among users of On-site pharmacy:** OR 6.0 (95% CI 1.18–31.0), *p* = 0.03
Duininck et al. [31]	2019	Multi-disciplinary team	R	Pre- and post-intervention	Patients diagnosed with HCC	**Increased referrals to surgery:** 49% vs. 30%; *p* = 0.02 **Increased liver-directed therapy:** 58% vs. 31%; *p* = 0.001 **More likely to get treatment:** 59% vs. 41%; *p* = 0.026 **Improved median OS:** 30.7 vs. 4.9 months; *p* < 0.001
Jain et al. [68]	2019	Formal Education + EHR Alert	R	Pre- and post-intervention	Providers and baby boomers	**Increased HCV screening:** 10.1% (pre) vs. 34.6% (post), *p* < 0.0001
Singal et al. [75]	2019	Mailed Outreach +/− Patient Navigation	P	RCT	Patients with cirrhosis	**Higher rates of HCC surveillance over 18 month period:** - Mailed outreach + navigation vs. usual care: 23.3% vs. 7.3%, *p* < 0.001 - Mailed outreach alone vs. usual care: 17.8% vs. 7.3%, *p* < 0.001 - Mailed outreach + navigation vs. mailed outreach alone: 23.3% vs. 17.8%, *p* = 0.02
Wong et al. [69]	2020	Formal Education	P	RCT	Patients with chronic HBV in liver clinic	**Increased rates of clinic follow up:** 81.4% (education) vs. 39.2% (control); OR 7.02; 95% CI 3.64–13.56, *p* < 0.001 **Increased rates of appropriate lab monitoring:** 77.5% (education) vs. 42.2% (control). OR, 4.94; 95% CI, 2.64–9.24; *p* < 0.001
Desai et al. [76]	2021	Mailed Outreach	P	RCT	Baby boomers	**Increased rates of HCV screening:** in-reach + outreach vs. in-reach alone at - 3 months: 14.6% vs. 7.4%, *p* < 0.001 - 6 months: 17.4% vs. 9.8%, *p* < 0.001
Lee et al. [77]	2022	Patient Navigation	P	Descriptive and non-comparative	Patients with advanced fibrosis/cirrhosis	**High rates of screening:** 94%, 75%, and 74% of patients completed their first, second, and third imaging tests.
Singal et al. [78]	2022	Mailed Outreach	P	RCT	Patients with cirrhosis	**Higher semi-annual surveillance in outreach group:** 35.1% vs. 21.9%, *p* < 0.001 **Lower no-surveillance in outreach group:** 29.8% vs. 43.5%, *p* < 0.001
Strebe et al. [79]	2023	Patient Navigation	R	Pre- and post-intervention	Baby boomers who tested positive for HCV	**Higher odds of linkage to care at 6 months:** OR, 3.7; 95% CI, 2.9–4.8 **Treatment initiation within 6 months:** OR, 3.2; 95% CI, 2.3–4.2 **No difference in SVR:** 86.9% vs. 86.1%; *p* = 0.78

P = prospective; R = retrospective; RCT = randomized controlled trial.

## Abbreviations

ACA, Affordable Care Act; ALD, alcohol-associated liver disease; CI, confidence interval; DAA, direct-acting anti-HCV; DSH, Disproportionate Share Hospital; EMR, electronic medical record; ER, emergency room; ESLD, end-stage liver disease; HBV, hepatitis B; HCC, hepatocellular carcinoma; HCUP, Healthcare Cost and Utilization Project; HCV, hepatitis C; LT, liver transplantation; MASH, metabolic dysfunction-associated steatohepatitis; MASLD, metabolic dysfunction-associated steatotic liver disease; ME, Medicaid expansion; NACSELD, North American Consortium for the Study of End-Stage Liver Disease; NHANES, National Health and Nutrition Examination Survey; OPTN, Organ Procurement and Transplantation Network; OR, odds ratio; PCP, primary care physician; SNH, safety-net hospital; SSI, Supplemental Security Income; SVR, sustained virologic response; UNOS, United Network for Organ Sharing; US, United States.
